# Mapping phosphoproteins in *Mycoplasma genitalium *and *Mycoplasma pneumoniae*

**DOI:** 10.1186/1471-2180-7-63

**Published:** 2007-07-02

**Authors:** Hsun-Cheng Su, Clyde A Hutchison, Morgan C Giddings

**Affiliations:** 1 Department of Microbiology and Immunology, University of North Carolina at Chapel Hill, Chapel Hill, NC 27599, USA; 2 J. Craig Venter Institute, Rockville, MD 20850, USA; 3 Joint Department of Biomedical Engineering, University of North Carolina at Chapel Hill, Chapel Hill, NC 27599, USA and North Carolina State University, Raleigh, NC 27695, USA; 4 Department of Computer Science, University of North Carolina at Chapel Hill, Chapel Hill, NC 27599, USA

## Abstract

**Background:**

Little is known regarding the extent or targets of phosphorylation in mycoplasmas, yet in many other bacterial species phosphorylation is known to play an important role in signaling and regulation of cellular processes. To determine the prevalence of phosphorylation in mycoplasmas, we examined the CHAPS-soluble protein fractions of *Mycoplasma genitalium *and *Mycoplasma pneumoniae *by two-dimensional gel electrophoresis (2-DE), using a combination of Pro-Q Diamond phosphoprotein stain and ^33^P labeling. Protein spots that were positive for phosphorylation were identified by peptide mass fingerprinting using MALDI-TOF-TOF mass spectrometry.

**Results:**

We identified a total of 24 distinct phosphoproteins, about 3% and 5% of the total protein complement in *M. pneumoniae *and *M. genitalium*, respectively, indicating that phosphorylation occurs with prevalence similar to many other bacterial species. Identified phosphoproteins include pyruvate dehydrogenase E1 alpha and beta subunits, enolase, heat shock proteins DnaK and GroEL, elongation factor Tu, cytadherence accessory protein HMW3, P65, and several hypothetical proteins. These proteins are involved in energy metabolism, carbohydrate metabolism, translation/transcription and cytadherence. Interestingly, fourteen of the 24 phosphoproteins we identified (58%) were previously reported as putatively associated with a cytoskeleton-like structure that is present in the mycoplasmas, indicating a potential regulatory role for phosphorylation in this structure.

**Conclusion:**

This study has shown that phosphorylation in mycoplasmas is comparable to that of other bacterial species. Our evidence supports a link between phosphorylation and cytadherence and/or a cytoskeleton-like structure, since over half of the proteins identified as phosphorylated have been previously associated with these functions. This opens the door to further research into the purposes and mechanisms of phosphorylation for mycoplasmas.

## Background

Post-translational modification of proteins is known to be a critical part of many biological pathways, yet in the mycoplasma species, little is known about the extent of protein modification that occurs. Mycoplasmas are wall-less prokaryotes and primarily mucosal pathogens of respiratory or urogenital tracts [1]. *Mycoplasma genitalium *has the smallest genome (580,070 bp) known for a free-living organism, with its close relative *Mycoplasma pneumoniae *having a larger genome (816,394 bp) that contains orthologs of all of the *M. genitalium *genes plus approximately 200 additional genes [2-4]. While only a few occurrences of phosphorylation, lipid modification, and proteolytic processing of proteins in the mycoplasmas have been characterized, rows of multiple spots with the same molecular weights have been observed on 2-D gels [5,6]. This suggests that there are yet to be discovered post-translational modifications, but the mechanisms and targets are generally unknown. Since phosphorylation is a common modification in other bacterial species, yet only three confirmed cases have been shown in these mycoplasma species [5,7], we used proteomic methods to examine the prevalence and targets of phosphorylation in *M. genitalium *and *M. pneumoniae*.

Bacteria commonly utilize three different phosphorylating systems to modify their proteins [8]. The first is an ATP/GTP-dependent protein kinase system, which leads to the phosphorylation of serine, threonine, and tyrosine residues. The second type is a 'two-component system', which includes a histidine kinase protein that receives a signal and transmits it to a partner response regulator protein through its phosphorylation. The response-regulator protein in turn transmits the signal to a downstream target. The third is the phosphoenol pyruvate:carbohydrate phosphotransferase system (PTS). The genomes from *M. genitalium *and *M. pneumoniae *encode no known two-component systems [9], which has contributed to a viewpoint that phosphorylation might be less frequent in the mycoplasmas than in bacteria having two-component systems. Two phosphorylated proteins previously verified in *M. pneumoniae *are the cytadherence-associate proteins HMW1 and HMW2, phosphorylated at threonine and serine residues in an ATP-dependent manner [5,10]. Both of them are members of a specialized attachment tip structure at one pole in *M. pneumoniae*, called the complex terminal organelle. More recently, the phosphocarrier protein Hpr [7], a component of the phosphoenol pyruvate:carbohydrate phosphotransferase system, was discovered.

We examined the CHAPS-soluble phosphoproteome of *M. genitalium *and *M. pneumoniae*, applying a combination of fluorescence-multiplexed 2-D gel electrophoresis [11] with Pro-Q Diamond staining, ^33^P labeling detected by autoradiography [12], and MALDI-TOF-TOF mass spectrometry (MS). The Pro-Q Diamond dye is a mass spectrometry compatible small-molecule fluorophore that permits rapid and direct detection of phosphoproteins [13] by binding exclusively to the phosphate groups. Since it is a new technology, we verified the Pro-Q results by comparing them to ^33^P labeling. We also compared cells in stationary phase versus exponential growth in order to determine the effect that nutrient limitation has on phosphorylation of proteins in the mycoplasmas.

## Results and Discussion

We identified 24 distinct proteins that are phosphorylated in the mycoplasmas, 22 in *M. genitalium *and 18 in *M. pneumoniae*, by combining the results of 2D gel electrophoresis analysis of soluble proteins using both the Pro-Q Diamond stain and ^33^P labeling.

### Fluorescence-based analysis of phosphoproteins of mycoplasmas

**Figure 1:**
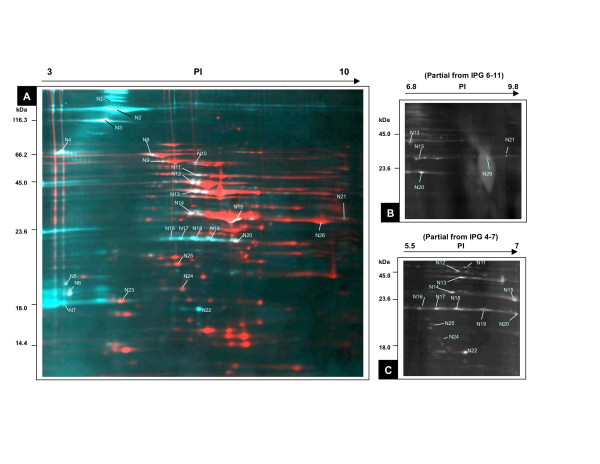
**CHAPS-Soluble Phosphoprotein map for *Mycoplasma pneumoniae***. 250 μg CHAPS-soluble cellular extract of stationary phase *M. pneumoniae *proteins were separated by 2D PAGE using 12.5%T SDS-polyacrylamide gels. **(A) **Shows a gel separated in the first dimension using a linear pH gradient **p*I*3–10 **(18 cm IPG strip). Identified phosphorylated proteins are numbered (see Table 3 for details). Molecular weight markers are indicated at the left and p*I *values at the top. The gel was stained with Pro-Q Diamond for detection of phosphoproteins (shown as blue/white), scanned, and the same gel was then stained with SYPRO Ruby protein stain (shown as red). The overlay images were generated using ImageMaster 2D software. [56] **(B) **Shows a partial image of an IPG 6–11 gel stained by Pro-Q Diamond, and **(C) **shows a partial image from an IPG 4–7 gel stained by Pro-Q Diamond.

The primary method used to assay phosphorylation in both *M. pneumoniae *and *M. genitalium *was the Pro-Q diamond stain. We used 2D PAGE to separate soluble proteins derived from each of these species at both exponential growth phase and stationary phase, staining the gels using Pro-Q Diamond to detect the phosphorylated fraction then SYPRO Ruby to detect the total protein complement. The computer-generated overlays of the images for *M. pneumoniae *proteins obtained at stationary phase are shown in Figure 1, where the Pro-Q detected phosphoproteins are shown as blue/white spots and the SYPRO Ruby stained spots are shown in red. The ratio of the Pro-Q Diamond dye signal to the SYPRO Ruby dye signal (here abbreviated as D/S ratio) was used as an approximate measure of the level of phosphorylation with respect to the amount of each protein in a sample [13]. In some cases the strong SYPRO Ruby signal made it difficult to discern the overlaid Pro-Q signal (e.g. spot N26), so panels B and C of Figure 1 show the Pro-Q signal for gels with smaller PI ranges (6–11 and 4–7, respectively) without a SYPRO Ruby overlay.

**Figure 2:**
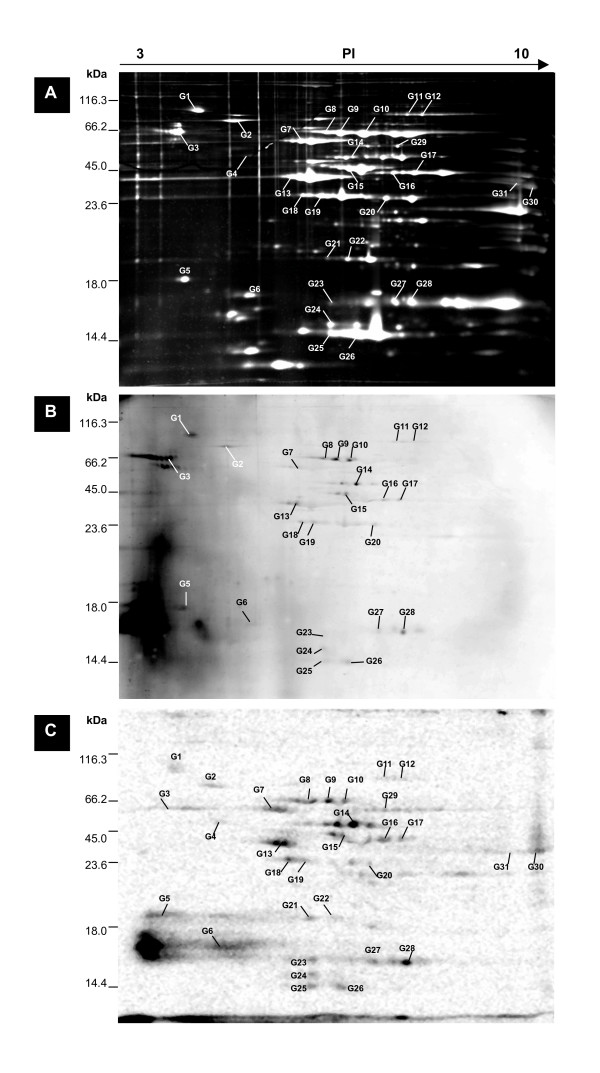
**CHAPS-Soluble Phosphoprotein map for *Mycoplasma genitalium***. 250 μg CHAPS-soluble cellular extract of stationary phase *M. genitalium *proteins were separated by 2-D gel electrophoresis with immobilized linear pH gradients from 3 to 10 and 12.5%T SDS-polyacrylamide gels. Panel **(A) **shows the 2-D gel with the total protein complement stained by SYPRO Ruby. Molecular weight markers are indicated at the left, and p*I *values at the top. Panel **(B) **shows the proteins stained by Pro-Q Diamond, and **(C) **shows the autoradiographic gel obtained by [^33^P]-phosphoric acid-labeling. Identified phosphorylated proteins are numbered (see Table 2 for details).

Figure 2 shows the results for *M. genitalium *in three separate panels, the top being the total protein stain by SYPRO Ruby, the middle being for the Pro-Q Diamond stained proteins, and the bottom for the ^33^P labeled proteins.

Combining the results from 2-D gel electrophoresis with IPG gradients of 3–10, 4–7 and 6–11, approximately 230 protein spots (for *M. genitalium*) and 310 protein spots (for *M. pneumoniae*) were observed with the SYPRO Ruby stain. There were about 30 protein spots in each strain reproducibly detected by the Pro-Q Diamond stain, with many of these apparently due to multiple isoforms of the single phosphoproteins. A few protein spots stained intensely with Pro-Q Diamond but not with SYPRO Ruby, which may have resulted from proteins with multiple phosphorylation sites, resulting in high-sensitivity detection by Pro-Q Diamond.

Thirty-one of the labeled spots from the *M. genitalium *gels (Figure 2), and 20 spots from *M. pneumoniae *(Figure 1), were selected for MALDI-TOF-TOF mass spectrometry (MS) identification. Six spots from the *M. pneumoniae *gel, that stained as phosphoproteins by Pro-Q Diamond, were directly identified by correspondence to a previously published 2D proteome map, so mass spectrometry was not performed on these spots [6,14]. To confirm the correspondence mapping, we also determined that the identity of the protein was the same in each case as for the corresponding *M. genitalium *protein we identified by our own mass spectrometry efforts.

A total of 24 distinct proteins were identified between both strains, with an example peptide mass fingerprint-based identification shown in Table 1. There were 22 distinct phosphoproteins identified in *M. genitalium *(Table 2), with eight proteins presenting multiple spots due to different isoforms present. In *M. pneumoniae*, 12 independent proteins were identified by MS and in addition to the six identified based on correspondence to identified spots in the previously published gel (Table 3). All but two of the analyzed proteins had one or more isoforms with significant (p < 0.05) Mascot protein identification scores [15]. For the two *M. pneumoniae *spots N22 and N26 not producing protein identification scores above the significance threshold, we located phosphorylated homologs with significant scores in the *M. genitalium *gels. Of the 51 spots analyzed, only spot N16 did not produce any Mascot identification results. Based on its position in a row of spots with the same molecular weight (N17, N18, N19, and N20), it may be an isoform of the hypothetical protein (H10_orf220L) that was identified in these other spots.

**Table 1:** Example: matching the *M. genitalium *spot G18 peptide mass fingerprint data with protein B64230 in the database

Start-End	Observed *m/z*	*M*_r _(expt)	*M*_r _(calc)	Delta mass(Da)	Modification	Missed cleavages	Database sequence	Peptide sequence ion score
4–22	2157.10	2156.09	2156.09	-0.00	Oxidation (M)	0	K.IQVNNIEALNNAMDLALER.D	25
23–41	2070.99	2069.98	2069.98	0.00		0	R.DQNVVLYGQDAGFEGGVFR.A	105
109–118	1074.65	1073.65	1073.62	0.02		0	R.GVYTAPLVVR.M	37
147–157	1268.59	1267.59	1267.58	0.01	Oxidation (M)	0	K.TVMPSNPYDTK.G	-
158–176	2090.11	2089.11	2089.11	-0.01		0	K.GLFLAAIESPDPVIFFEPK.K	-
158–177	2298.19	2297.19	2297.17	0.01	Phospho (ST)	1	K.GLFLAAIESPDPVIFFEPKK.L	-
230–239	1171.69	1170.69	1170.66	0.03		1	K.DKGIELIDLR.T	-
232–239	928.57	927.56	927.54	0.02		0	K.GIELIDLR.T	45
247–254	922.51	921.51	921.49	0.01		0	K.QTVFNSVK.K	-
259–267	971.63	970.62	970.61	0.01		0	R.LLVVTEAVK.S	42
294–305	1286.77	1285.76	1285.74	0.02		0	R.VTGFDIVVPLAR.G	72
309–316	1040.54	1039.53	1039.51	0.02		0	K.YQFEINAR.V	52
317–326	1112.68	1111.67	1111.66	0.01		0	R.VIDAVNQLLK.-	41

The peptide profile of spot G18, treated with trypsin and analyzed by MALDI-TOF-TOF. Mascot was used for the database search with MSDB version 20050227. 13/45 peptides were matched to B64230, pyruvate dehydrogenase E1-beta chain. The peptide sequence ion score corresponds to peptides analyzed and identified by MS/MS sequencing, and peptides without a score (-) were detected only in the MS data. Ion scores of 20 or greater are significant. The matched peptide sequences are shown by underline in the sequence below.

1   MSKIQVNNIEALNNAMDLALERDQNVVLYGQDAGFEGGVFRATKGLQQKY GSERVWDCPI AENSMAGIGV GAAIGGLKPI VEIQFSGFSF PAMFQIFVHA

101 ARIRNRSRGVYTAPLVVRMP MGGGIKALEH HSETLEAIYA QIAGLKTVMPSNPYDTKGLFLAAIESPDPVIFFEPKKLYR AFRQEIPSDY YTVPIGEANL

201 ISEGSELTIV SYGPTMFDLI NLVYSGELKDKGIELIDLRT ISPWDKQTVFNSVKKTGRLLVVTEAVKSFT TSAEIITSVT EELFTYLKKA PQRVTGFDIV

301 VPLARGEKYQFEINARVIDAVNQLLK

**Table 2:** Identified phosphoproteins of *M. genitalium*

Spot	Protein name	Gene ID; Protein ID & Locus tag	Theoretical p*I/M_r_*	Peptide mass fingerprinting		
				
				Match peptides ^a)^	Sequence coverage coverage (%)	MS & MS/MS score ^b)^
G1	Putative structural protein involved in cytoskeleton	875488; C64236 (MG328)	4.61/88353.9	26 (1)	39	811
G2	Heat shock protein *dna*J homolog MG200	875694; A64222 (MG200)	4.86/68494.8	17 (2)	30	396
G3	P65 protein	875361; I64223 (MG217)	4.59/44636.8	17 (1)	33	464
G4 ^c)^	Conserved hypothetical protein	875359; AAC71439 (MG218.1)	4.96/39701.4	17(3)	51	461
G5	DNA-directed RNA polymerase delta chain	875707; D64202 (MG022)	4.48/17046.3	6 (0)	55	204
G6	Transcription elongation factor *gre*A homolog	875338; B64231 (MG282)	4.99/18151.5	13 (1)	81	857
G7	Heat shock protein GroEL	875662; D64243 (MG392)	5.82/58318.4	37 (2)	67	1030

G8	DnaK-type molecular chaperone	875435; G64233; (MG305)	6.79/65010.5	28 (0)	46	997
G9				29 (1)	44	1120
G10				26 (2)	45	1160

G11	Cytadherence-accessory protein	875571; A64235	7.01/	22 (2)	40	401
G12	(hmw3) homolog	(MG317)	68679.1	24 (2)	42	515

G13	Pyruvate dehydrogenase E1-alpha chain	875404; C64230 (MG274)	5.55/40625.3	13 (1)	45	651
G14	Phosphopyruvate hydratase -(enolase)	875644; A64245 (MG407)	6.63/49987.9	15 (2)	45	297
G15	Translation elongation factor EF-Tu	875605; EFYMTG (MG451)	6.18/42963.3	14 (2)	44	582

G16	Restriction-modification enzyme	875615; D64248	7.58/	26 (6)	52	770
G17	EcoD specificity chain	(MG438)	44082.8	23 (6)	46	409

G18	Pyruvate dehydrogenase E1-beta	875399; B64230	6.04/	13 (1)	41	697
G19	chain	(MG273)	36002.8	13 (1)	41	749

G20	Phosphate acetyltransferase	875564; A64233 (MG299)	6.97/35446.5	13 (0)	47	575

G21 ^c)^	Hypothetical protein	875676; G64241 (MG377	6.24/22635	27(5)	74	864
G22 ^c)^				20(3)	66	642

G23	Methionine sulfoxide reductase A	875645; B64245 (MG408)	6.18/18403.2	6 (1)	33	147

G24	Conserved hypothetical protein	875626; B64247 (MG427)	6.19/	7(1)	82	220
G25			15593	4(0)	30	
G26				7(1)	82	

G27	Hypothetical protein MG342	875477; H64237 (MG342)	8.37/	8 (1)	56	248
G28			19115.9	4 (1)	30	82

G29 ^c)^	ATP synthase alpha chain	875650; (MG401)	6.70/57215.4	22(6)	55	220
G30 ^c)^	L-lactate dehydrogenase and	875596; (MG460)	8.76/33966.2	28(1)	72	566 ^d)^
	Ribosomal protein S2	875215 (MG070)	9.36/32746.8	17(4)	46	
G31 ^c)^	L-lactate dehydrogenase	875596; (MG460)	8.76/33966.2	25(3)	70	396

a) Number of peptides that match the theoretical digest of the primary protein identified. The number in parentheses is the count of potentially phosphorylated peptides found by Mascot and FindMod (see additional file 1: Putative_Phosphopeptides.pdf, Table A).

b) Combined score for the peptide mass fingerprint including both MS and MS/MS peptide matches for the spectrum. Scores of 95 or greater were considered significant (p < 0.05).

c) Identified proteins detected by labeling with ^33^P-phosphoric acid.

d) Spot G30 was identified to contain both L-lactate dehydrogenase and Ribosomal protein S2. The MS & MS/MS score shown here belongs to the L-lactate dehydrogenase.

**Table 3:** Identified phosphoproteins of *M. pneumoniae*

Spot	Protein name	Gene ID; Protein ID & Locus tag	Theoretical p*I/M*_r_	Peptide mass fingerprinting		
				
				Match a) peptides	Sequence coverage (%)	MS & MS/MS score ^b)^
N1	Putative structural protein	877396; S73693	4.94/	9 (1)	9	99
N2	involved in cytoskeleton (MG328 homolog)	(MPN474)	118006.7	22 (2)	24	357

N3	Cytadherence accessory protein HMW3	877094; S73715 (MPN452)	4.67/73676.1	22 (1)	42	883
N4^c)^	P65	876932; S73853 (MPN309)	4.27/47009.1	-	-	-

N5	Probable DNA-directed RNA	876869;	4.32/	4 (1)	20	112
N6	polymerase delta subunit	RPOE_MYCP	17163.2	3 (0)	15	33
N7		(MPN024)		9 (1)	57	325

N8^c)^	DnaK-type molecular chaperone	876838; S73733 (MPN434)	5.41/65091.4	-	-	-
N9^c)^	Heat shock protein GroEL	876804; S73595 (MPN573)	5.61/58082.8	-	-	-
N10	Trigger factor tig	876961; S73831 (MPN331)	6.10/51321.1	15 (1)	37	544
N11	Phosphopyruvate hydratase – (enolase)	876984; S73562 (MPN606)	6.12/49197.4	10 (1)	25	179
N12	Translation elongation factor EF-Tu	877030; S73503 (MPN665)	6.06/43122.3	21 (3)	55	664
N13	Pyruvate dehydrogenase E1-alpha chain	877083; S73771 (MPN393)	6.11/40568.4	19 (0)	63	491

N14	Pyruvate dehydrogenase	877125; S73772	6.47/	9 (2)	36	221
N15	E1-beta chain	(MPN392)	35891.6	15 (1)	52	733

N16	No Significant Hits	**-**	**-**	**-**	**-**	**-**

N17	Hypothetical protein	876832; S73866	7.66/	9 (1)	34	123
N18	H10_orf220L	(MPN295)	25684.2	8 (1)	35	57
N19				9 (2)	33	115
N20				16 (2)	60	244

N21	Ribosomal protein S2	876805; S73949 (MPN208)	9.16/33404.7	20 (1)	48	487
N22	MG427 homolog (Osmotical inducible protein C like family)	877343; S73543 (MPN625)	6.50/15458.9	5 (1)	45	84
N23 ^c)^	Transcription elongation factor	877111; S73763 (MPN401)	4.91/18101.5	-	-	-
N24 ^c)^	Methionine sulfoxide reductaseA	876738; S73561 (MPN607)	5.64/18378.6	-	-	-
N25 ^c)^	Hypothetical protein	877212; S73613 (MPN555)	5.58/22433.8	-	-	-
N26	L-lactate dehydrogenase	877032; S73494 (MPN674)	8.35/33888.1	12 (1)	52	56

a) Number of peptides that match the theoretical digest of the primary protein identified. The number in parentheses is the count of potentially phosphorylated peptides found by Mascot and FindMod (see additional file 1: Putative_Phosphopeptides.pdf, Table B).

b) Combined score for the peptide mass fingerprint including both MS and MS/MS peptide matches for the spectrum. Scores of 95 or greater were considered significant (p < 0.05).

c) Protein spots N4, N8, N9, and N23~ N25 were identified by previous existing proteome maps [6, 14]; "-" means that MS analysis was not performed for these spots. The MS data for N16 was of insufficient quality for identification.

The 22 phosphorylated proteins detected in *M. genitalium *represent about five percent of the 480 *M. genitalium *ORFs [2,16], whereas the 18 identified in *M. pneumoniae *represent about three percent of the 688 predicted *M. pneumoniae *ORFs. However, since not all expressed proteins were observed in the gels, this is likely an underestimate. Also, since the *M. pneumoniae *strain we used is attenuated and lacks P90 and P40, the phosphorylation patterns might be altered from wild-type. A more realistic number might be obtained from the ratio of phosphoproteins observed to total proteins observed on the gels, which is 9% (22/230) for *M. genitalium *and 6% (18/310) for *M. pneumoniae*. For comparison, *E. coli *has over 130 phosphoproteins, which is about three percent of its annotated ORFs [17], and *Corynebacterium glutamicum *also has three percent of its cytoplasmic proteins phosphorylated [12]. Although the mechanisms of and targets for phosphorylation are different between these species, the ratio of proteins phosphorylated is quite consistent, adding evidence that regardless of bacterial species, protein phosphorylation plays an important signaling role in the cell.

Twenty-two of the phosphoproteins we identified had not been previously reported in the mycoplasmas. There were eight phosphoproteins that occurred in only one of the strains (Table 4), six from *M. genitalium *and two from *M. pneumoniae*. Since *M. pneumoniae *contains a strict superset of orthologs to *M. genitalium *[4], a case such as MPN295 (an *M. pneumoniae *specific product) only detected in the former (spots N17-N20) is not surprising [18]. For the other phosphoproteins only detected in one strain, it is possible that the ortholog in the other species is also phosphorylated, but that we did not detect it.

**Table 4:** Functional categories of phosphoproteins, the variation of protein phosphorylation levels, phosphoprotein orthologs, and the potential association with cytoskeleton-like structure in mycoplasmas

	Proposed function/annotation	MG, MPN number	Stat/Exp growth variation ^a)^	Phosphorylated in both *M. genitalium *and *M. pneumoniae *^b)^	In Triton-X100 insoluble fraction ^c)^
Energy metabolism	Pyruvate dehydrogenase E1-beta chain (PdhB)	MG273 MPN392	↑	Y	+
	Pyruvate dehydrogenase E1-alpha chain (PdhA)	MG274 MPN393	↑	Y	+
	L-Lactate dehydrogenase	MG460 MPN674	-	Y	+
	ATP synthase alpha chain	MG401	-	N	×
	Phosphotransacetylase	MG299	-	N	+
Carbohydrate transport and metabolism genes	Phosphopyruvate hydratase (enolase)	MG407 MPN606	-	Y	×
Translation/transcription	Elongation factor Tu (Tuf)	MG451 MPN665	↑	Y	+
	DNA-directed RNA polymerase delta subunit	MG022 MPN024	↓	Y	×
	Trigger factor (Tig)	MPN331	↑	N	+
	Ribosomal protein S2	MG070 MPN208	-	Y	×
	Transcription elongation factor (GreA)	MG282 MPN401	-	Y	+
Heat shock, chaperones	Heat-shock protein GroEL	MG392 MPN573	↑	Y	+
	Heat-shock protein DnaK	MG305 MPN434	↑	Y	+
	Heat-shock protein DnaJ homolog	MG200	-	N	×
Cytaherance-associated proteins	Cytadherance accessory protein (HMW3)	MG317 MPN452	↓	Y	+
	Protein 65	MG217 MPN309	↑	Y	+
	Putative structural protein involved in cytoskeleton	MG328 MPN474	↑	Y	+
Other functions	Restriction-modification enzyme EcoD	MG438	↓	N	×
	Methionine sulfoxide reductase A	MG408 MPN607	-	Y	×
Unknown functions	Osmotic inducible protein-C-like family.	MG427 MPN625	↑	Y	+
	Hypothetical protein	MG377 MPN555	-	Y	×
	Hypothetical protein H10_orf220L.	MPN295	↑	N	+
	Hypothetical protein	MG342	↑	N	×
	Conserved hypothetical protein	MG218.1	-	N	×

a) The variation of the Pro-Q Diamond dye signals (D)/SYPRO Ruby dye (S) signals between bacteria in stationary phase versus exponential growth phase. "↑" means the phosphorylation level was enhanced in stationary phase, "↓" means the phosphorylation level decreased in stationary phase, and "-"means no significant change was observed.

b) "Y" means the phosphoprotein was detected in both *M. genitalium *and *M. pneumoniae*; "N" means the corresponding protein was not identified in both strains.

c) "+" means the detected phosphoprotein was part of a Triton-X100 insoluble fraction putatively associated with the cytoskeleton-like structure as reported in [18], and "x" means it was not.

We also examined the MS analyzed peptides matched by Mascot and FindMod, and in all but six of the identified proteins, at least one peptide mass was found that matches a predicted phosphopeptide for the protein (see additional file 1: Putative_Phosphopeptides.pdf for a list of all predictions). Most of these corresponded with low-intensity peaks, as is typically the case for phosphopeptides due to poor ionization efficiency in the presence of phosphoryl groups [19].

### Comparison of Pro-Q fluorescence detection with *in vivo *radiolabeling

Pro-Q Diamond is a relatively new method for rapid and global protein phosphorylation analysis in gels [13], which may overcome some of the challenges with ^33^P labeling or immunodetection with antibodies, such as cross-reactivity in immunodetection and the difficulty of obtaining the right growth conditions for radiolabeling [20]. Because of limited reports of its use in the literature, we used ^33^P to perform *M. genitalium in vivo *radiolabeling of phosphoproteins for comparison with and verification of the Pro-Q Diamond stain results. The generation time of *M. genitalium *is generally 12–16 h, so for the radiolabeling experiments we incubated 24 h for full uptake of ^33^P by cells. Figure 2B shows the Pro-Q Diamond stained control sample grown under identical conditions, and Figure 2C shows the radioactive phosphoproteins detected by scanning the dried gel after exposure to storage phosphor screen (Amersham) for 3 days. We found approximately 30 protein spots by each of the two methods, and most of the spots are well matched between the two detection methods. However, spots G4, G21, G22, G29, G30 and G31 were seen only on the radiolabelled gels, and spot G3 was stained by Pro-Q Diamond but not observed by radiolabelling. Possible reasons for the differences in detection include: (i) insufficient accessibility to the site of phosphorylation in some proteins for the Pro-Q Diamond dye, for example due to a steric hindrance effect; (ii) the presence of phospho-enzyme intermediates that can only be detected by metabolic radiolabeling, for example when phosphoglucomutase forms phosphoseryl enzyme intermediates during the catalytic cycle [21] they are detected by radiolabeling but not immunostaining [12]; (iii) *in vivo *isotope labeling may alter cell cycle progression and morphology, potentially influencing the modification state of proteins [22,23]. Despite these few differences, the > 80 % agreement between the methods indicates that Pro-Q Diamond dye is accurate for detection of phosphoproteins, and has the advantage of speed and simplicity. Most importantly, the radiolabelling adds supporting evidence for phosphorylation of most of our reported phosphoproteins.

### Biological categorization of identified phosphoproteins

We functionally categorized the identified phosphoproteins using Regula *et al*. [18] and COGs [24,25] as guides, as shown in Table 4. Phosphoproteins we identified are involved in energy metabolism, carbohydrate transport, carbohydrate metabolism, translation, transcription, heat shock response/chaperoning, cytadherence, and other unknown functions. Interestingly, fourteen of our identified phosphoproteins had previously been associated with the cytoskeleton-like structure in *M. pneumoniae *[18]. These proteins include pyruvate dehydrogenase E1 alpha and beta chains, elongation factor Tu, heat shock protein GroEL, DnaK, cytadherence accessory proteins, trigger factor, osmotic inducible protein-C-like family and hypothetical protein H10_orf 220L.

Several proteins we identified were previously shown as phosphorylated in other prokaryotic cells, including phosphopyruvate hydratase (enolase), ATP synthase, and RNA polymerase [12,26]. Ribosomal protein S2 was identified as a phosphoprotein in both strains (spots N21 and G30), though it co-separated with a phosphorylated isoform of L-lactate dehydrogenase in *M. genitalium*, causing concern that the phosphorylation could be on the latter protein. However, in *M. pneumoniae*, these two spots were resolved separately, and the S2 spot remained stained by Pro-Q Diamond. Though S2 has not previously been reported as phosphorylated, there is some precedent for ribosomal protein phosphorylation, with S1 being phosphorylated in *E. coli *[27,28]. Also, the NetPhos WWW server[29,30] predicted 10 strong phosphorylation sites on S2, one of which matched a detected MALDI peak corresponding to the putative phosphopeptide "ENLML**S**R", where there was a high score for predicted phosphorylation on the serine residue (Ser-153).

### Estimated phosphorylation levels in different growth states

**Figure 3:**
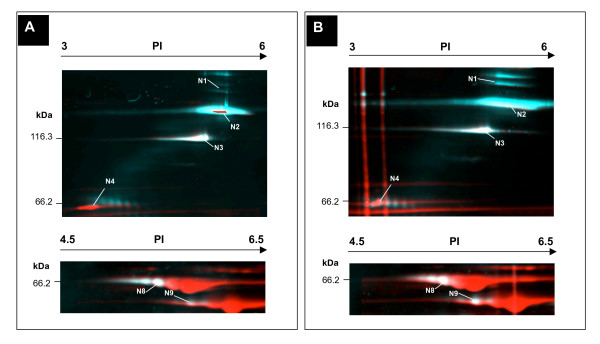
**Comparison of protein phosphorylation between different growth states**. Selected sections of the overlay image from the 2D-gel phosphoprotein profile of **(A) **exponential growth phase and **(B) **stationary growth phase of *M. pneumoniae *are shown, with growth cycle determined by wet cell weight as described in the methods. Phosphoproteins were detected by Pro-Q Diamond stain (shown as blue/white) and then the total proteins were labeled by SYPRO Ruby protein stain (shown as red). See Figure 1 and Table 3 for protein numbering and details. A putative structural protein (spots N1 and N2), and heat shock protein GroEL (spot N9) displayed a significant rise in protein phosphorylation when *M. pneumonia*e was harvested for analysis in the stationary phase.

As cells enter the stationary growth phase, nutrients become depleted and inter-cellular competition is increased. In *E. coli *and *Thiobacillus ferrooxidans*, phosphorylation of the heat shock proteins DnaK and GroEL was shown to be modulated by environmental stresses including temperature, nutrient starvation and pH change [31]. To determine whether there was a similar effect upon phosphorylation in the mycoplasmas, we compared protein abundances for samples obtained during stationary growth phase to those from cells harvested at exponential growth phase by comparing the Pro-Q Diamond (D) to SYPRO Ruby (S) ratio between growth conditions (Table 4, 3^rd ^column: Stat/Exp growth variation). While there was an expected reduction in overall protein synthesis for stationary phase growth [32], the phosphoproteins DnaK, GroEL (Figure 3, see spot N8, N9), Elongation factor Tu and the pyruvate dehydrogenase complex were up-regulated in both species. In addition, putative structural protein MPN474, heat shock protein DnaK, GroEL, P65 (Figure 3, spots N1, N2, N8, N9, and N4 from *M. pneumoniae*), elongation factor Tu and pyruvate dehydrogenase all appeared to have increased phosphorylation levels during stationary phase. The phosphorylation level of HMW3 in both *M. genitalium *and *M. pneumoniae *(Figure 3, spot N3 from *M. pneumoniae*) was slightly reduced during stationary growth, as were the restriction modification enzyme EcoD and the DNA-directed RNA polymerase delta subunit. The increase in phosphorylation during stationary phase for mycoplasmas involves some of the same proteins as the response in *E. coli *(DnaK and GroEL), pointing at a role for these proteins in stress sensing and response.

### Protein phosphorylation in mycoplasma cytadherence and cytoskeleton-like proteins

Both *M. pneumoniae *and *M. genitalium *have a complex terminal organelle structure that enables them to adhere to a host cell for colonization and nutrient acquisition [33-35]. The terminal organelle is a membrane-bound extension of the cell, characterized by an electron-dense core comprised of rod-like structures oriented laterally within the tip [36], and thin fibrous structures extending into the cell body. The proteins P1, HMW1, HMW2, HMW3, P90, P40, P30 and P65 were found to be localized to the complex terminal organelle by a variety of techniques such as immunofluorescence microscopy [33,37-39], with P1 playing a major receptor-binding role in *M. pneumoniae *[9,40,41], and the others also shown necessary for cytadherence [33,36-38]. Of these cytadherence proteins, HMW1 and HMW2 were found to be phosphorylated, and there was weaker evidence that P1 may also be phosphorylated [5]. We found two more cytadherence proteins phosphorylated in both species studied, HMW3 (spots N3, G11, and G12), and P65 (spots G3, N4). Adding these to the previous identifications, at least four out of the eight cytadherence-critical proteins appear to be phosphorylated, indicating an important role for phosphorylation in the process of host-cell attachment.

We did not detect HMW1, HMW2, P30, and P40/P90, corroborating a previous report that these proteins were not found when IEF was used for separation in the first dimension. In previous studies, HMW1 [5] and P40/P90 [42] were found using NEPHGE (Non-Equilibrium pH Gradient Electrophoresis) instead of IEF for the first dimension. And HMW2 (M_r _≈ 216,000), which has the tendency to form multimeric complexes, has only been identified using a 1-D polyacrylamide gel [6]. A faint signal was detected in the SYPRO Ruby stain for P1 indicating its presence in the gel, but it was not detected by Pro-Q Diamond or radiolabeling, giving no evidence for its phosphorylation, unlike the previous report. The recently reported phosphoprotein Hpr, was also not detected. It was previously reported that this protein was phosphorylated in the presence of glycerol [7], so our culture medium may not have promoted its phosphorylation.

These cytadherence-related proteins, except P30, were also detected in a study of the Triton X-100 insoluble protein fraction from *M. pneumoniae *[18]. As in eukaryotic cells [43], it is thought that the Triton X-100 insoluble proteins comprise cytoskeleton-like structural elements, of which the complex terminal organelle is a part. We found a number of the other reported Triton X-100 insoluble mycoplasma proteins to be phosphorylated, including pyruvate dehydrogenase E1, elongation factor Tu, heat shock proteins DnaK and GroEL. Of the 24 phosphorylated proteins identified in our study, fourteen (58%) were reported as Triton X-100 insoluble.

Further supporting these associations with cytadherence and a cytoskeleton-like structure, it was previously demonstrated that elongation factor Tu and pyruvate dehydrogenase E1 beta are expressed on the *M. pneumoniae *cell surface and involved in binding to eukaryotic cell surfaces [44]. In *E. coli*, EF-Tu functions as a translation/transcription protein, but it has also been observed to polymerize to form filaments and bundles with actin-like properties [45], indicating that it is likely to play more than one role in bacteria. DnaK, GroEl, enolase and elongation factor EF-Tu have been reported as phosphorylated in *Corynebacterium glutamicum*, *Thiobacillus ferrooxidans *and *E. coli*, respectively [12,31,46].

We also found a high molecular weight (118 kDa) putative structural protein to be phosphorylated in *M. pneumoniae*, MPN474 (spots N1, N2 in Fig.1), along with a homologous protein (88 kDa), MG328 (spot G1 in Fig. 2), in *M. genitalium*. This protein was found in the Triton X-100 insoluble fraction, and has been annotated as "coiled coil putative structural protein involved in cytoskeleton" like HMW2, which is typical of filamentous domains of known cytoskeletal proteins [47]. According to a protein motif scan by "MyHit" [48], these proteins contain several leucine zipper motifs as is the case in HMW2, providing potential for dimerization interactions. Given all the evidence, it appears that this protein may be important in the formation of a cytoskeleton-like structure.

The combination of previous results and our own indicate that a significant fraction of the mycoplasma cytadherence and cytoskeleton-like proteins are phosphorylated. This may be analogous to eukaryotic cells where phosphorylation and dephosphorylation of cytoskeletal elements has been associated with changes in cell morphology [49].

In the studies by Seto and Miyata [38] as well as Kenri *et al*. [39], the locations of the proteins correlated with cytadherence in *M. pneumoniae *were determined. The electron-dense core in the attachment organelle of *M. pneumoniae *is thought to be the same as a rod-like structure comprised of cytadherence-related proteins (including HMW1, HMW2, and HMW3) that was observed in mycoplasma cells treated with detergent [18]. The wheel-like structure resembles a flagellar motor, and may be connected to the filamentous network extending into the cytoplasm of *M. pneumoniae *cells. While the composition of the filamentous network [50] is not definitively known, several of the proteins identified as phosphorylated in this study show evidence of being involved in the formation of filamentous bundles, including EF-Tu, DnaK, and pyruvate dehydrogenase[41,45].

**Figure 4:**
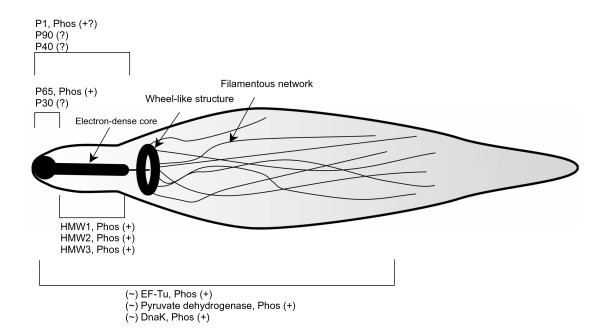
**Schematic of phosphorylated proteins within *Mycoplasma pneumoniae *overlaid on a diagram of the hypothesized complex terminal organelle and cytoskeleton-like structure**. "Phos (+)" means the protein has been identified as phosphorylated in our data or previous studies. "Phos (+?)" means there is weak experimental evidence published supporting phosphorylation, and "(?)" means there is no evidence regarding phosphorylation, but that the protein was localized by immunofluorescence studies to the cytoskeleton-like structure. All protein locations are represented based upon the presently available literature data, but may be subject to change based upon further studies. For proteins labeled with "(~)", conflicting evidence indicates that they may have multiple locations in the cell. The localization of proteins were determined as follows: HMW1, HMW2 and HMW3 were shown essential in the electron-dense core of the terminal organelle in *M. pneumoniae*, though HMW3 may also have other cellular locations; P30 and P65 are localized at the surface of the distal end of the terminal organelle [38, 39]; P1, P90 and P40 are known to interact, with P1 penetrating the cell membrane, anchored to cytoskeletal structures by P90 and P40 [41]; and in a cross-linked protein complex with the P1 adhesin of *M. pneumoniae*, it appears that DnaK might be involved in translocation of proteins from cytoplasm to the membrane. Pyruvate dehydrogenase is implicated as a structural protein in the attachment organelle [41]. EF-Tu is a putative cytoskeletal element in *E. coli *[45], but has also been associated with translation so it may have multiple functions and hence locations. It, along with DnaK and pyruvate dehydrogenase, have previously been shown involved in the filamentous network. [41, 45]

We combined these lines of evidence into Figure 4, representing the prevailing hypotheses and data regarding the cystoskeleton-like structural elements in the mycoplasmas, with the phosphorylation data overlaid. While the figure is speculative due to the fact that the cellular location of the elements represented have not all been definitively pinpointed, there is a strong overall trend apparent in the association of phosphorylated proteins with the cytoskeleton-like structure. We hypothesize that this correlation is because phosphorylation plays an important role in the formation and/or regulation of the structure, and that it may represent a signaling network in the cell. Whether or not the hypothesis proves true, this evidence warrants further study regarding the functional role of phosphorylation in host attachment, stress response, and cell morphology.

## Conclusion

Though it was previously not known whether phosphorylation was prevalent or played a significant role in the mycoplasmas, our results indicate that phosphorylation occurs at rates comparable to other studied prokaryotic species. Based on the functional categories of the phosphoproteins found, protein phosphorylation in the mycoplasmas may be involved in sensing and regulation of stress responses, as well as cell division, gliding motility and cytadherence.

## Methods

### Materials and equipment

Mycoplasma Broth Base (BBL), Bacto Tryptone (Difco), Bacto peptone (Difco) Yeastolate 4% (Difco), and Tissue culture flasks, Corning Costar and Nalgene (25 cm^2 ^and 185 cm^2^) were from Fisher Scientific Co. (Agawam, MA, USA). CMRL 1066 1 × TC medium and Yeast Extract solution were from Gibco-Invitrogen (Carlsbad, CA, USA). Fetal Bovine Serum, Penicillin G, L-glutamine, Phenol red solution (0.5%), CHAPS, iodoacetamide and DTT were from Sigma-Aldrich (St. Louis, MO, USA). The IPGphor™ for IEF, ready-made IPG DryStrips (pH4–7, 3–10, 6–11), 2-D Pharmalyte pH 3–10, IPG buffer and ImageQuant software for image analysis were from Amersham Biosciences (Piscataway, NJ, USA). Pefabloc SC was from Roche (Mannheim, Germany). The Protean II xi 2-D cell was from Bio-Rad (Richmond, CA, USA). Pro-Q Diamond phosphoprotein and SYPRO Ruby Protein gel stain were from Invitrogen-Molecular Probes (Invitrogen-Molecular Probes, Carlsbad, CA, USA). The 2D-Gel imaging system, ProXPRESS, was from PerkinElmer (Boston, MA, USA), and the ABI 4700 MALDI TOF/TOF mass spectrometer was from Applied Biosystems (Foster, CA, USA).

### Bacterial strains media and growth conditions

*Mycoplasma pneumoniae *M129-B170 (ATCC 29343), a broth-passage attenuated strain that lacks P90 and P40 [42], and *Mycoplasma genitalium *G37 (ATCC 33530) were grown in SP-4 medium at 37°C as in [51] except that 10^3 ^U/ml penicillin was used, and L-glutamine was added separately. The cells were grown in 25 cm^2 ^tissue culture flasks containing 10 ml SP-4 medium. Cultures were harvested as soon as the medium turned orange. The cells were resuspended in 2 ml 50% trypticase soy broth and 50% fetal bovine serum solution via pipetting for the seed cultures. 185 cm^2 ^tissue culture flasks containing 120 ml SP-4 medium were inoculated with 20 μl fresh prepared seed culture. Cultures were harvested with a cell scraper followed by centrifugation when the cells were in exponential phase (72 h *M. pneumoniae*; 120 h *M. genitalium *cultures) and stationary phase (96 h *M. pneumoniae*; 144 h *M. genitalium *cultures).

### Protein extraction

Extracts of *M. genitalium *and *M. pneumoniae *proteins were prepared essentially as described [6] with minor modifications. The washed cells were resuspended in 600 μl lysis solution containing 9 M urea, 2% (w/v) CHAPS, 1% (w/v) DTT, 2% (v/v) pharmalyte (pH3-10) and 2 mM Pefabloc SC. Cells were disrupted with a Branson sonifier for 1 min 30 sec (duty cycle: 40, output control: 3) at 4°C. Cell debris and protein aggregates were removed by centrifugation at 80,000 × g for 60 min in a Beckman L5-50B ultracentrifuge (Fullerton, CA, USA). The protein concentration was determined using the Bradford method [52] with the Protein Assay kit (Bio-Rad, Hercules, CA, USA). The supernatants containing the proteins were split into aliquots and frozen at -78°C.

### Two-dimensional separation of proteins

About 250 μg of protein from whole-cell extracts of *M. genitalium *and *M. pneumoniae *was analyzed using the O'Farrell gel technique [53]. Before 2-D gel electrophoresis, all protein samples were precipitated following the ProQ Diamond Stain manufacturer's recommendation (Molecular Probes Inc.), to minimize nonspecific staining due to phospholipids and other cell constituents. The protein precipitates were dissolved and loaded by the in-gel rehydration method for 13 h in the lysis solution mentioned above (except using different IPG buffers of pH 4–7 and 6–11 suitable for IPG strips). Isoelectric focusing was performed with commercially available IPG-strips (Amersham Bioscience; pH 3–10, 4–7, 6–11) and run on an IPGphore unit (Amersham Bioscience). Voltage profiles were as follows: 30 V for 390 Vh, 1000 V for 1000 Vh, 2000 V for 4000 Vh, 4000 V for 6000 Vh, 6000 V for 12000 Vh, 8000 V for 14000 Vh, with a total focusing of 38 kVh. Prior to the second dimension, strips were equilibrated as the described by Görg *et al*. [54,55]. After completion, 12.5% large-format SDS-polyacryl-amide gel electrophoresis was performed using a Protean II xi 2-D cell (Bio-Rad).

### Multiplexed fluorescence detection and image analysis

Fluorescent staining of 2-D SDS-polyacrylamide gels was performed using Pro-Q Diamond phosphoprotein stain and SYPRO Ruby protein stain (Invitrogen-Molecular Probes, Carlsbad, CA, USA) following the manufacturer's instructions. All steps of staining and washing were performed using slow agitation on an orbital mixer (50 rpm). As a control to test the selectivity of the Pro-Q Diamond stain for phosphoproteins, PeppermintStick phosphoprotein markers (Invitrogen-Molecular Probes, Carlsbad, CA, USA), a standard with two phosphorylated and four nonphosphorylated proteins, were loaded at approximately 500 ng/each protein on SDS poly-acrylamide gel. After electrophoresis the gels were stained with Pro-Q Diamond phosphoprotein stain and imaged, then stained with SYPRO Ruby and re-imaged. Images were acquired on a ProXPRESS Proteomic Imaging System (PerkinElmer, Boston, MA, USA), with 550 nm excitation and 580 nm bandpass emission filters used following Pro-Q Diamond dye staining, and 480 nm excitation and 620 nm bandpass emission filters used following SYPRO Ruby dye staining. Gel images were analysed by ImageQuant (Amersham Bioscience, Piscataway, NJ, USA), and the differential display maps of protein phosphorylation and protein expression patterns were generated using ImageMaster 2D software, in which we imported TIFF image files, annotated the selected protein spots as the landmarks, and then selected and stacked gels to obtain an alignment using transparency [56].

### *In vivo *^33^P -labeling analysis of mycoplasmas proteins

Freshly prepared *M. genitalium *seed culture (20 μl) was inoculated into two 75-cm^2 ^tissue culture flasks, each containing 25 ml of SP4 medium and harvested at exponential growth. The cells in one of these flasks were labeled with ^33^P-phosphoric acid by a method similar to [5], except that the cells were grown in SP4 medium. The second flask was used as a control. Briefly, for the ^33^P labeled cells, the SP4 medium was removed from *M. genitalium *cultures in the 5^th ^day (during exponential growth), and they were washed three times with Tris-saline (0.15 M NaCl, 20 mM Tris-HCl [pH 7.5]). SP4 was replaced with 10 ml of MegaCell Dulbecco's phosphate free Modified Eagle's Medium (Sigma, St. Louis, MO, USA), supplemented with the nonessential amino acids alanine, asparagine, aspartic acid, glutamic acid, glycine, proline and serine, in addition to 20 mM HEPES. The re-suspended cultures were transferred to a new tissue culture flask for labeling. H_3_^33^PO_4 _(Amersham Biosciences, Piscataway, NJ, USA) at 2500 Ci/mmol, was added to obtain a final culture concentration of 30 μCi/ml, with incubation continued for 24 h at 37°C. The cells were harvested and washed with Tris-saline and used immediately for protein sample preparation. The control culture was identically processed, except that unlabelled phosphoric acid (H_3_PO_4_) was added instead of H_3_^33^PO_4_.

For each culture, isoelectric focusing was performed on a 7 cm IPG strip (pH 3–10, Amersham Biosciences) using an IPGphore unit (Amersham Biosciences), with 50 μg of radio-labeled protein in a DeStreak rehydration solution (Amersham Biosciences, Piscataway, NJ, USA). The second dimension separation was performed by mini SDS-PAGE electrophoresis. 2D gels were vacuum-dried on Whatman paper (Florham Park, NJ, USA), and scanned to obtain total protein profiles. The gel containing the ^33^P labeled proteins was exposed to a storage phosphor screen (Amersham Biosciences) for 3 days, then scanned with Typhoon 9400 (Amersham Biosciences).

### In-gel digestion

In-gel tryptic digestion and mass spectrometry were performed at the University of North Carolina Michael Hooker Proteomics Core Facility. The phosphoprotein spots were picked with a 2D-iD robotic spot picker from Leap Technologies (Carrboro, NC, USA), into 96 well micro-titre plates for automated proteolytic digestion on an Investigator ProGest robot (Genomic Solutions, Ann Arbor, MI, USA). The proteolytic digestion was performed according to the established protocols of the University of North Carolina Proteomics Core Facility [57].

### MALDI-TOF-TOF MS and database searches

The peptide extracts were used for MALDI-TOF-TOF protein identification. MALDI mass spectra were obtained with an ABI4700 Proteomics Analyzer (TOF/TOF; Applied Biosystems Inc. Foster City, CA, USA). The system obtained peptide masses, and the top ten mass peaks above a signal-to-noise ratio of 35 were automatically selected for MS/MS analysis, producing combined MS and MS/MS data for the peptide mass fingerprint. The resulting data were searched against the Mass Spectrometry Protein Sequence Database (MSDB), a composite non-identical protein sequence database, using the search engine Mascot [58]. Two missed cleavages per peptide were allowed and a mass tolerance of ± 80 ppm was used in all searches. Partial oxidation for methionine and phosphorylation for serine, threonine and tyrosine were assumed. We also used the FindMod tool [59] and the NetPhos WWW server [29,30] to verify the observed data we got from Mascot. In FindMod, we set the search to look for one potential post-translational modification per peptide with ± 80 ppm mass tolerance, allowing for up to two missed cleavage sites. For phosphorylation site prediction with NetPhos, we entered the identified protein sequence in FASTA format for prediction of phosphorylation sites at tyrosine, serine and threonine residues.

## Abbreviations

DTT dithiothreitol

IEF isoelectric focusing

IPG immobilized pH gradient

MALDI-TOF-TOF matrix-assisted laser desorption/ionization time of flight tandem mass

## Authors' contributions

HCS, CAH and MCG participated in the design of the study. HCS performed the experiment, data analysis and drafted the manuscript. CAH and MCG coordinated the study and provided extensive suggestions on the research as it progressed. MCG revised and edited the final manuscript. All authors read and approved the final manuscript.

## Supplementary Material

Additional file 1 Summary of Potentially Phosphorylated Peptides. Two tables, A and B, listing all potentially phosphorylated peptides matching the mass spectrum PMF peptide masses, for *M. genitalium *(Table A) and *M. pneumoniae *(Table B). Predictions combined from *Mascot *[58] and *FindMod *[59]. Click here for file
