# Supplementary material for: Mapping phosphoproteins in Mycoplasma genitalium and Mycoplasma pneumoniae
Source: BMC Microbiol. 2007 Jul 2;7:63. doi: 10.1186/1471-2180-7-63 (PMC1947986; doi:10.1186/1471-2180-7-63)
Supplement: Additional file 1 — Summary of Potentially Phosphorylated Peptides. Two tables, A and B, listing all potentially phosphorylated peptides matching the mass spectrum PMF peptide masses, for M. genitalium (Table A) and M. pneumoniae (Table B). Predictions combined from Mascot [58] and FindMod [59]. [file 1471-2180-7-63-S1.pdf]

Summary of potentially phosphorylated peptides

Table A. Predicted phosphopeptides in *M. genitalium* based on MALDI-MS masses

Protein name (Locus tag)	Peptide sequence Start- End	Observed *m/z*[1]	M_r (expt)[1]	M_r (calc)[1]	Delta mass (Da)[1]	Modification[2]	Missed cleavages	Phosphopeptide sequence[3]
Putative structural protein involved in cytoskeleton (MG328)	740-748	1215.63	1214.62	1214.54	0.08	1 Phospho (Y)	1	ERIQAYAER
Heat shock protein *dna*J homolog MG200 (MG200)	445-458	1484.71	1483.71	1483.60	0.11	1 Phoshpo (STYHCD)	1	GFGNDHGKGCGDLK
	506-525	2284.18	2283.18	2283.17	0.01	1 Phospho (STYHCD)	2	VDGEIDINAIMKFEKLGIAK
P65 protein (MG217)	282-287	856.53	885.52	885.40	0.12	1 Phospho (ST)	1	MQKLTR
Conserved hypothetical protein (MG218.1)	29-53	2808.30	2807.30	2807.40	-0.09	1 Phospho (ST)	1	QLNELKQILVSLDNQEASATAVTDK
	35-54	2211.10	2210.09	2210.08	0.01	1 Phospho (ST)	1	QILVSLDNQEASATAVTDKK
Transcription elongation factor *gre*A homolog (MG282)	104-122	2239.14	2238.13	2238.02	0.11	1 Phospho (Y)	1	SNEKYTIVGTLEANPEEHK
Heat shock protein GroEL (MG392)	13-27	1718.86	1717.85	1717.96	-0.11	1 Phospho (ST)	2	TRLLQGINKIANAVK
	508-515	969.5	968.5	968.49	0.00	1 Phospho (ST)	1	VTKTALEK
DnaK-type molecular chaperone (MG305)	38-47	1311.62	1310.61	1310.57	0.04	1 Phospho (ST), 1 Phospho (Y)	1	RTTPSIVSYK
	39-59	2596.19	2595.18	2595.08	0.10	1 Phospho (ST), 1 Phospho (Y)	2	TTPSIVSYKNNEIIVGDAAKR
Cytadherence-accessory protein (hmw3) homolog (MG317)	39-57	2283.18	2282.17	2282.07	0.10	1 Phospho (Y)	1	VHALYQDPESGNIFSLEKR
	387-415	3337.66	3336.65	3336.69	-0.03	3 Phospho (ST), 1 Phospho (Y)	2	TEYQPQQPLPTSGLQIKVVPRSAALLQSK
Pyruvate dehydrogenase E1-alpha chain (MG274)	259-282	2843.34	2842.34	2842.21	0.12	1 Phospho (Y)	2	QGPHTTSDDPSIYRTKQEEEEGMK

Phosphopyruvate hydratase -(enolase) (MG407)	35-60	2692.26	2691.25	2691.26	-0.01	1 Phospho (ST),	1	LASGHVGEAMVPSGASTGEKEAIELR
	423-439	2211.11	2210.10	2210.08	0.02	2 Phospho (Y)	2	IAKYNRLLYIEIELGDK
Translation elongation factor EF-Tu (MG451)	167-177	1176.57	1175.56	1175.65	-0.04	2 Phospho (ST)	0	NTPIIYGSALK
	286-304	2181.11	2180.10	2180.18	-0.08	1 Phospho (ST)	2	EVERGQVLAKPGSIKPHKK
Restriction-modification enzyme EcoD specificity chain (MG438)	27-49	2807.29	2806.28	2806.22	0.06	2 Phospho(Y)	2	GEMLEKELITPEGKYEYFNGGVK
	33-40	966.51	965.50	965.45	0.05	1 Phospho (ST)	0	ELITPEGK
	57-80	2749.29	2748.29	2748.27	0.01	1 Phospho (ST), 1 Phospho (Y)	2	FNTFKNTISVIVGGSCGYVRLADK
	354-368	1875.90	1874.89	1874.95	-0.06	2 Phospho (ST)	2	ELSSLTVIRDTLLKK
	363-376	1802.94	1801.93	1801.87	0.06	Oxid. (M), 1 Phospho(ST)	2	DTLLKKLFPDMTER
	363-376	1882.89	1881.89	1881.83	0.05	Oxid. (M), 2 Phospho(ST)	2	DTLLKKLFPDMTER
Pyruvate dehydrogenase E1-beta chain (MG273)	158-177	2298.19	2297.19	2297.17	0.02	1 Phospho (ST)	1	GLFLAAIESPDPVIFFEPKK
Hypothetical protein (MG377)	2-19	2299.18	2298.17	2298.08	0.09	2 Phospho (ST), 1 Phospho (Y)	2	ATNLKSIAKLQKPIQYDK
	46-62	2207.11	2206.11	2205.95	0.16	2 Phospho (ST), 1 Phospho (Y)	1	DAKESALYHELTHIVIK
	105-116	1451.72	1450.71	1450.82	-0.11	1 Phospho (ST)	2	TIQSIAEKIIKK
	126-138	1682.85	1681.84	1681.81	0.03	1 Phospho (ST)	2	EWKVEVSDDIVKR
	138-146	1330.66	1329.65	1329.58	0.07	2 Phospho (Y)	1	RVISLYYEK
	153-165	1118.53	1117.53	1117.47	0.06	1 Phospho (Y)	1	EYLDDKQK
Methionine sulfoxide reductase A (MG408)	112-117	856.53	855.52	855.45	0.07	1 Phospho (STYHCD)	0	FLKLQK
Conserved hypothetical protein (MG427)	110-132	2808.31	2807.30	2807.23	0.07	2 Phospho (ST)	2	EFIDFVSKRCPAHNTLHGTSNFK

Hypothetical protein (MG342)	22-39	2239.13	2238.14	2238.12	0.02	1 Phospho (STYHCD)	2	KFANELKHSLSCELIELK (Cysteine treated with Iodoacetamide to form carbamidomethyl-cysteine).
ATP synthase alpha chain (MG401)	14-21	1076.54	1075.53	1075.53	0.00	1 Phospho (Y)	2	TEIKKYSK
	23-40	1940.95	1939.94	1939.96	0.02	1 Phospho (ST)	0	IFNSEIGQVISVADGIAK
	141-164	2807.30	2806.30	2806.31	-0.01	3 Phospho (ST)	1	SVNQPLETGILTIDALFPIGKGQR
	172-187	1794.83	1793.83	1793.93	-0.10	1 Phospho (ST)	1	QTGKTAIAIDTIINQK
	279-296	2261.11	2260.11	2260.00	0.11	1 Phospho (ST), 1 Phospho (Y)	1	RPPGREAFPGDVFYLHSR
	460-472	1598.85	1597.84	1597.71	0.13	1 Phospho (ST)	0	ITEEFNGSHPLFK
L-lactate dehydrogenase (MG460)	6-25	2225.12	2224.12	2223.94	0.17	3 Phospho (ST)	0	IAIVGSGAVGTSFLYAAMTR
	3-25	2507.26	2506.25	2506.11	0.13	3 Phospho (ST)	0	GPKIAIVGSGAVGTSFLYAAMTR
Ribosomal protein S2 (MG070)	106-123	2233.09	2232.09	2232.00	0.08	3 Phospho (ST)	1	WLGGTLTNFKTLSISINK
	116-126	1310.70	1309.69	1309.70	-0.01	1 Phospho(ST)	1	TLSISINKLNK
	124-140	2063.91	2062.90	2063.04	-0.14	1 Phospho(ST)	2	LNKLVEQQKQNANDLTK
	133-148	2017.06	2016.05	2015.94	0.11	2 Phospho (ST)	2	QNANDLTKKENLLLSR

1. Observed *m/z* is calculated as the experimental molecular mass (Da) plus the molecular mass (Da) of any additional protons, divided by the charge given by the protons. Mr(expt) is the experimental molecular mass of the peptide (Da). Mr(calc) is the calculated molecular mass of the peptide (Da). Delta mass is given by Mr(expt) - Mr(calc) (Da).
2. Potentially phosphorylated peptides were predicted by a search using both Mascot and FindMod (details provided in the methods section, subhead: “MALDI-TOF-TOF MS and database searches”). For Mascot, the following variable modifications were searched: Oxidation (M), Phospho (ST) and Phospho (Y); and for “FindMod” search, the variable modifications searched were: Oxidation (M), Phospho (ST), Phospho(Y), Phospho(H), Phospho(C) and Phospho(D). The number listed for each entry shows how many phosphorylation sites were predicted for each peptide.
3. Potential phosphorylation sites underlined in the peptide sequence. The potential phosphopeptide of “methionine sulfoxide reductase A” was not localized by the prediction.

Table B. Predicted phosphopeptides of *M. pneumoniae* based on MALDI-MS masses

Protein name (Locus tag)	Peptide sequence Start- End	Observed *m/z*[1]	M_r (expt)[1]	M_r (calc)[1]	Delta mass (Da)[1]	Modification[2]	Missed cleavages	Phosphopeptides sequence[3]
Putative structural protein involved in cytoskeleton (MPN474)	537-545	1312.60	1311.59	1311.49	0.10	1 Phospho (ST), 1 Phospho (Y)	1	LNDENTKYR
	550-561	1602.72	1601.71	1601.58	0.13	Oxid. (M), 1 Phospho (ST), 1 Phospho (Y)	1	QYELMRADSDAK
Cytadherence accessory protein HMW3 (MPN452)	652-670	2239.14	2238.13	2238.14	-0.01	1 Phospho (ST)	3	SPLRGGLSQRFPLRSSWSK
Probable DNA-directed RNA polymerase delta subunit (MPN024)	14-21	1045.53	1044.53	1044.45	0.07	1 Phospho (ST)	0	EIAETQFK
Trigger factor tig (MPN331)	3-14	1559.81	1558.80	1558.78	0.03	1 Phospho (Y)	2	QYKLVNTTQKEK
Phosphopyruvate hydratase - (enolase) (MPN606)	1-11	1278.56	1277.55	1277.54	0.01	1 Phospho (ST)	0	MSAQTGTDLFK
Translation elongation factor EF-Tu (MPN665)	39-52	1661.79	1660.78	1660.75	0.04	1 Phospho (Y)	2	EGKSAATRYDQIDK
	58-74	2087.95	2086.94	2086.82	0.12	2 Phospho (ST), 1 Phospho (Y)	1	ARGITINSAHVEYSSDK
	58-75	2083.97	2082.96	2082.98	-0.02	1 Phospho (Y)	2	ARGITINSAHVEYSSDKR
Pyruvate dehydrogenase E1-beta chain (MPN392)	290-306	1929.04	1928.03	1928.00	0.03	1 Phospho (ST)	1	AAPQRVTGWDIVVPLAR
Hypothetical protein H10_orf220L (MPN295)	168-175	1126.53	1125.52	1125.59	-0.08	1 Phospho (Y)	1	KLYLRPEK
	118-124	941.55	940.55	940.42	0.13	1 Phospho (ST)	1	QSWKNAK

Ribosomal protein S2 (MPN208)	148-154	942.46	941.45	941.40	0.05	1 Phospho (ST)	0	ENLML<u>S</u>R
MG427 homolog (Osmotical inducible protein C like family) (MPN625)	65-79	1802.91	1801.91	1801.88	0.02	2 Oxid. (M), 1 Phospho (ST)	1	MI<u>T</u>INKLLMNVTG<u>S</u>R
L-lactate dehydrogenase (MPN674)	190-200	1290.64	1289.63	1289.69	-0.06	1 Phospho (ST)	1	IAGLPLKHF<u>S</u>K

1. Observed *m/z* is calculated as the experimental molecular mass (Da) plus the molecular mass (Da) of any additional protons, divided by the charge given by the protons. Mr(expt) is the experimental molecular mass of the peptide (Da). Mr(calc) is the calculated molecular mass of the peptide (Da). Delta mass is given by Mr(expt) - Mr(calc) (Da).
2. Potentially phosphorylated peptides were predicted by a search using both Mascot and FindMod (details provided in the methods section, subhead: “MALDI-TOF-TOF MS and database searches”). For Mascot, the following variable modifications were searched: Oxidation (M), Phospho (ST) and Phospho (Y); and for “FindMod” search, the variable modifications searched were: Oxidation (M), Phospho (ST), Phospho(Y), Phospho(H), Phospho(C) and Phospho(D). The number listed for each entry shows how many phosphorylation sites were predicted for each peptide.
3. Potential phosphorylation sites underlined in the peptide sequence.
